# Effects on general pain perception and dental pulp sensibility in probable sleep bruxism subjects by experimentally induced pain in a pilot study

**DOI:** 10.1038/s41598-023-33019-z

**Published:** 2023-04-10

**Authors:** Michelle Alicia Ommerborn, Adem Özbek, Maike Grunwald, Rita Antonia Depprich, Nicole Pascale Walentek, Michael Franken, Ralf Schäfer

**Affiliations:** 1 Department of Operative Dentistry, Periodontology, and Endodontology, Faculty of Medicine, Heinrich-Heine-University, Moorenstr. 5, 40225 Düsseldorf, Germany; 2 Department of Cranio- and Maxillofacial Surgery, Faculty of Medicine, Heinrich-Heine-University, Düsseldorf, Germany; 3 Clinical Institute of Psychosomatic Medicine and Psychotherapy, Faculty of Medicine, Heinrich-Heine-University, Düsseldorf, Germany

**Keywords:** Bruxism, Pain, Oral-health-related quality of life

## Abstract

In this pilot study, the general pain perception and the dental pulp sensibility of probable sleep bruxism (SB) subjects were compared with that of non-SB subjects. The cold pressor test (CPT), electric pulp test (EPT), and thermal pulp test with CO_2_ snow were executed by one trained dentist (blind to SB diagnosis). A one-factorial multivariate analysis of variance (MANOVA) with SB diagnosis as independent variable and standardized measures regarding pain perception and evaluation was performed. One-hundred-and-five participants (53 SB and 52 non-SB subjects) were included. The one-factorial MANOVA revealed a significant difference between SB and non-SB subjects (*p* = 0.01) concerning pain perception variables. Post-hoc univariate analyses of variance (ANOVA) showed statistically significant lower general pain tolerance (*p* = 0.02), higher general subjective sensibility of the teeth (*p* < 0.01), and a statistical trend for higher subjective dental pain intensity (*p* = 0.07) in SB subjects. In most of the standardized variables, probable SB subjects seem to react and feel similar to non-SB subjects. However, as probable SB subjects subjectively perceive their teeth to be more sensitive and tend to rate their subjective dental pain intensity more intensely after CO_2_ testing, data might point to a somatosensory amplification.

## Introduction

Sleep bruxism (SB) has been defined as masticatory muscle activity during sleep that is characterized as rhythmic (phasic) or non-rhythmic (tonic) and is not a movement disorder or a sleep disorder in otherwise healthy individuals^1^. For the diagnosis of SB, it has been suggested that ‘possible’ SB should be based on a positive self-report, via questionnaires and/or the anamnestic part of a clinical examination. ‘Probable’ SB should be based on a positive clinical inspection, with or without a positive self-report and the inspection part of a clinical examination. ‘Definite’ SB should be based on positive instrumental assessment, like polysomnographic recordings, with or without a positive self-report or clinical inspection^1^. However, its timely, financial, and technical complexity hampers its routine use^2,3^.

Concerning its prevalence, the literature reports different results depending on the respective life stage. For example, SB activity reported by parents or sleep partners ranges from 14 to 17% in childhood, to about 8% in adulthood, and decreases to 3% in the elderly^4^.

The etiology of bruxism has a multifactorial character^5^. Many studies aimed to clarify the underlying mechanisms in the development of SB, which can be summarized to physiological/biological factors (e.g., neurotransmitters, genetics, sleep arousals)^6–12^, psychological factors (e.g., stress-related, predisposing personality traits, anxiety)^13–15^, and behavioral/pharmacological factors (e.g., tobacco, medications, drugs)^16^. Moreover, the possible relationship between SB and/or other types of parafunctional behavior, such as awake bruxism, and temporomandibular disorders (TMD) has long been a matter of scientific investigation^17–22^. As defined by the American Academy of Orofacial Pain, the term TMD comprises a group of musculoskeletal and neuromuscular conditions involving the temporomandibular joint (TMJ), the masticatory muscles, and all associated tissues^23^. The most frequent presenting symptom is pain, usually localized in the muscles of mastication and/or the preauricular area^24^.

Concerning the association of SB and pain, the literature provides only few information that is predominantly related to pain in the masseteric muscles^25,26^. Clinical investigations estimating the general pain perception in SB subjects by using standardized measures such as pain threshold, pain tolerance, and subjective pain intensity are still lacking. An altered general pain perception could be a predisposing factor for the development of painful TMD and, thus, a deeper exploration of the general pain perception in SB subjects would contribute to an increase in knowledge. Additionally, since the teeth act as a sort of effector organ of SB activity^27^ which has been induced by to date not yet clarified central mechanisms^5,28^, it would be interesting to assess the dental pulp sensibility in these subjects by using standardized measures such as pain threshold, pain duration, subjective pain intensity, and general subjective sensibility of the teeth, too. Typically, the pulp–dentin-complex is known to react to peripheral stimuli such as microorganism, attrition, erosion, periodontal diseases with the apposition of tubular sclerosis or reactionary dentin^29,30^. This reduced dentin permeability would lead to a lowered pulp sensibility. However, in a previous investigation evaluating non-carious cervical lesions (NCLs) in SB subjects, all subjects who had at least one NCL and at least one tooth with hypersensitivity were SB subjects without exception. None of the control subjects who had at least one NCL reported tooth hypersensitivity. Another explanation could be that SB subjects show an altered perception of pain stimuli because of increased levels of anxiety, depression, and stress^14,15,31^. The investigation of the general pain perception and the dental pulp sensibility in subjects with probable SB by using standardized measures would represent a novel approach that offers the opportunity of gathering new insights into the association between probable SB and TMD as well as its possible underlying mechanisms. In particular, the inclusion of psychological parameters and also aspects pertained to oral health-related quality of life could provide relevant contributions to the scientific community.

The aim of the pilot study was to examine, whether probable SB subjects perceive experimentally induced pain differently from non-SB subjects. Pain was induced by three procedures, the cold pressor test, the electric, and the thermal pulp test. Their application in SB subjects is not yet represented in science. It is therefore valid to consider the feasibility of the study protocol and initial effects^32,33^. The null hypotheses were that there would be no differences between SB and non-SB subjects in the intensity of (1) general pain perception and (2) dental pulp sensibility. Furthermore, the null hypothesis that there would be no difference in the degree of (3) psychological load between SB and non-SB subjects was tested.

## Results

### Demographic characteristics

**Figure 1:**
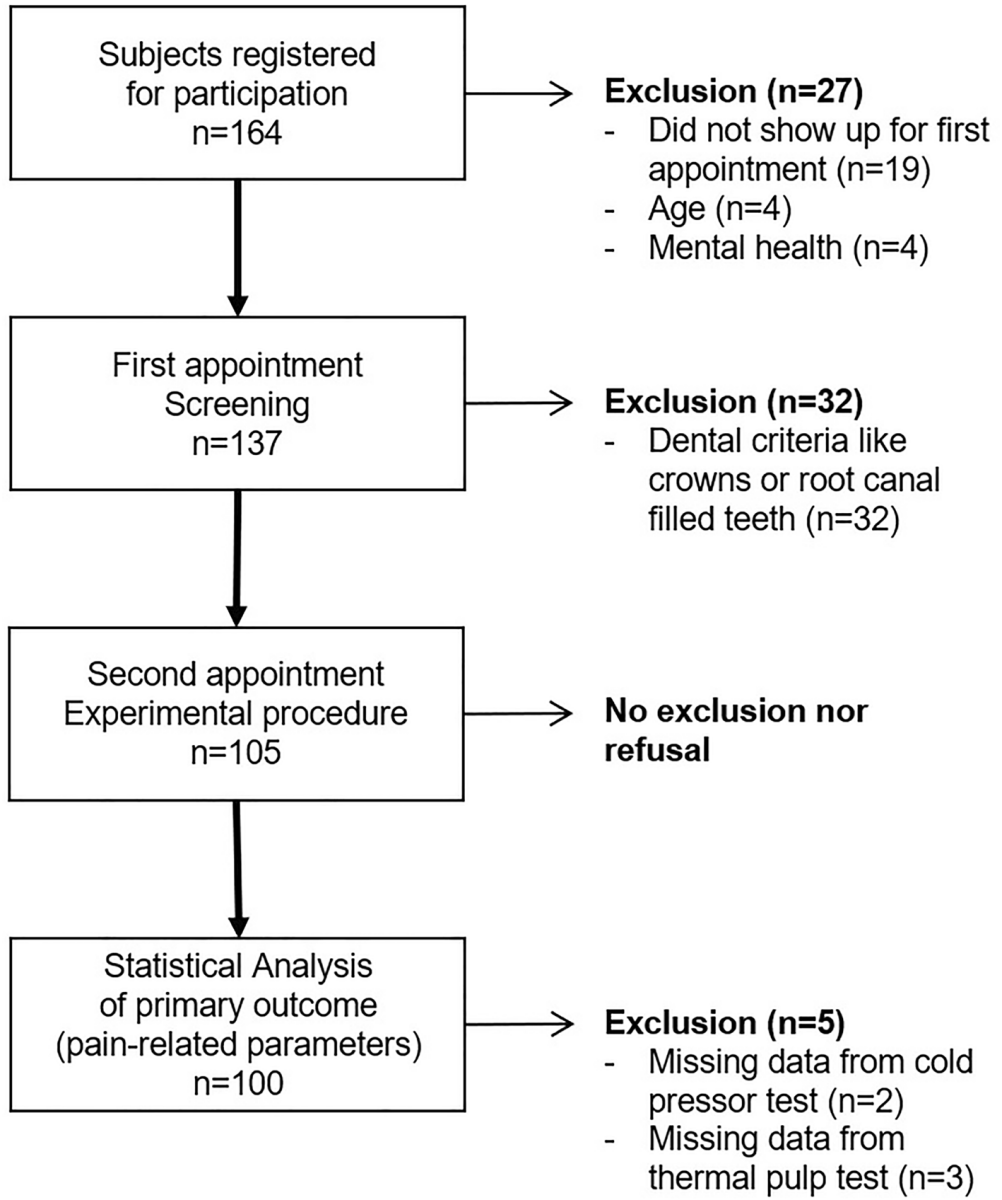
Flow-chart of the sample composition.

Figure 1 displays the flow of participants. The final sample consisted of *n* = 105 participants (63 females and 42 males, mean age ± SD: 29.90 ± 7.22 years). Table 1 displays sociodemographic data per group. There were no statistically significant group differences.

**Table 1:** Group differences in sociodemographic data (*n* = 105).

	SB subjects (*n* = 53)	Non-SB subjects (*n* = 52)	Statistic
Age^a^ (y)	29.57 ± 7.52	30.25 ± 6.95	W 1514.000; *p* = 0.38
Gender	37 F (35.24%)	26 F (24.76%)	*χ*^2^_*df* 1_ 3.507; *p* = 0.06
	16 M (15.24%)	26 M (24.76%)	
Education	12 H; 22 M; 19 L	17 H; 19 M; 15 L	*χ*^2^_*df* 2_ 1.51; *p* = 0.47

^a^Descriptive data is presented as M ± SD; *Y* years, *F* female, *M* male, *H* high educational level (at least college education), *M* middle educational level (at least high secondary education), *L* lower educational level, *df* degrees of freedom.

Regarding the portion of participants with a painful TMD diagnosis, the entire sample included approximately one third of participants with this diagnosis. A Chi-square test revealed significantly more painful TMD diagnoses in SB subjects *χ*^2^_*df* 1_ = 10.87, *p* < 0.01 (Table 2).

**Table 2:** Frequency distribution of painful TMD (*n* = 105).

	Painful TMD	Non-painful TMD (n = 4) + non-TMD (n = 68)
SB (n = 53)	25 (23.81%)	28 (26.67%)
Non-SB (n = 52)	8 (8.62%)	44 (41.90%)
Total	33 (31.43%)	72 (68.57%)

*SB* sleep bruxism, *TMD* temporomandibular disorder, *df* degrees of freedom.

### General pain perception and dental pulp sensibility

The one-factorial MANOVA with the SB diagnosis as the independent variable and the pain-related variables as dependent variables revealed a significant difference between SB and non-SB with *F*_(1,98)_ = 2.61, *p* = 0.01, partial *η*^2^_p_ = 0.18, Wilk’s Λ = 0.81. Participants with missing data (*n* = 5) in the variables general pain threshold, general pain tolerance (*n* = 2), and general subjective sensibility of the teeth (*n* = 3) were not included in the analysis. Table 3 displays the results of the post-hoc univariate ANOVAs. Of the eight pain-related variables, the general pain tolerance (*p* = 0.02) and the general subjective sensibility of the teeth (*p* < 0.01) differed significantly between the SB and the non-SB subjects. Moreover, a statistical trend for higher subjective dental pain intensity (*p* = 0.07) was observed.

**Table 3:** Post-hoc univariate ANOVAs, comparing pain-related outcome between SB and non-SB subjects (*n* = 100^a^).

Variable	SB subjects (*n* = 52)		Non-SB subjects (*n* = 48)		Statistic		
	Mean	SD	Mean	SD	*F*_(1,98)_	*P*	*η*^2^_p_
General pain threshold (s)	13.9	8.4	16.6	11.2	2.20	0.141	0.02
General pain tolerance (s)	21.5	12.2	28.6	19.4	5.78*	0.018	0.06
General subjective pain intensity (mm)	41.7	19.6	37.2	20.2	0.39	0.532	< 0.01
General subjective sensibility of the teeth (NRS)	4.2	3.1	2.4	2.6	8.48*	0.004	0.08
Number of EPT units	29.9	13.2	31.4	9.3	0.37	0.546	< 0.01
Dental pain threshold (s)	1.5	0.9	1.8	1.9	0.18	0.675	< 0.01
Dental pain duration (s)	4.9	2.3	4.3	2.6	2.40	0.125	0.02
Dental subjective pain intensity (mm)	55.9	24.9	45.7	24.9	3.27	0.074	0.03

*Statistically significant comparisons (*p* < 0.05).

*EPT* Electric Pulp Test, *NRS* Numeric Rating Scale from zero to ten, *SB* sleep bruxism.

^a^Missing data *n* = 5.

### Psychometric data

Based on non-normally distributed data from the GSI and the OHIP, a Mann–Whitney *U* test was computed. It revealed no significant difference between the SB (median = 0.34) and the non-SB group (median = 0.23) with W = 1108.50, *p* = 0.08. As the results of the analysis on the general pain perception and the dental pulp sensibility provide evidence of differences in subjective pain ratings, authors examined the subscale “somatization” of the SCL-90-R for statistical differences. There was a significant difference between the SB (median = 0.50) and the non-SB group (median = 0.33) with W = 1029.5, *p* = 0.03. The OHIP sum scores were also significantly different between the SB (median = 6) and non-SB group (median = 3) with W = 1060.50, *p* = 0.04. The median of the SB participants was located in the range of values obtained for patients with complete dentures in view of the aforementioned reference values.

Details about the descriptive data of the OHIP-G-14 domains are displayed in Table 4. Overall, SB show higher values in all domains compared to those of non-SB subjects. This implicates worse oral health-related quality of life in total, as well as in the specific domains.

**Table 4:** Descriptive data of OHIP-G-14 of SB and non-SB subjects (n = 105).

	SB subjects (n = 53)			Non-SB subjects (n = 52)		
OHIP-G-14 dimensions	Mean	SD	Median	Mean	SD	Median
Oral function	3.32	4.86	2	1.88	3.67	0
Orofacial pain	1.36	1.19	1	0.79	1.05	0
Orofacial appearance	1.04	1.33	0	0.60	0.98	0
Psychosocial impact	4.96	6.49	2	3.06	4.96	1
Total score	10.68	12.87	6	6.33	9.71	3

*SB* sleep bruxism.

### Feasibility of the of the applied procedures and the study conception

As displayed in Fig. 1, recruitment rate was with n = 164 subjects. Due to general exclusion criteria (16.46%) dental exclusion criteria (19.51%), and missing data (0.03%), data from n = 64 (39.02%) of the initial sample was not considered for statistical analysis (primary outcome). The introduction to the handling of the procedures was carried out by the study principal (M. A. O.) based on manuals and lasts 2 h. The first visit included an initial dental examination to check the dental inclusion and exclusion criteria, the verification of signs and symptoms of TMD and lasted 90 min. The actual performance of the pain-inducing procedures (CPT, 10 min; EPT, 10 min; thermal pulp test, 10 min) and the completion of the questionnaires (nearly 20 min) took approximately 50 min in total. Two trained dentists were assigned to the implementation. None of the participants discontinued the study during the experimental phase. 

## Discussion

Based on the results, the first null hypothesis, that there is no difference in general pain perception, could be partially rejected. SB subjects showed significantly lower pain tolerance than non-SB subjects. The second null hypothesis, that there is no difference in dental pulp sensibility, was also partially rejected. The SB subjects subjectively perceived their teeth as significantly more sensitive in general. In addition, they tended to rate their subjective pain intensity more intensely based on the CO_2_ test. Regarding psychological load, the last null hypothesis could be partially rejected, as the oral health-related quality was significantly worse in the SB subjects than in the non-SB subjects. Furthermore, the subscale “somatization” of the SCL-90-R^34^ revealed significantly higher scores in SB-subjects than in non-SB subjects.

Overall, the feasibility of the study can be rated as good, since the procedures could be performed independently and with no technical problems after a brief introduction. None of the subjects dropped out of the study, despite the aversive setting. The missing data in pain tolerance of the CPT are due to the fact that 2 subjects exceeded the maximum duration for the measurement of pain tolerance (> 5 min) and the tests were declared invalid.

To the authors’ knowledge, this is the first study in which the assessment of general pain perception and dental pulp sensibility is performed in one setting. Thus, the study design allowed direct comparison of pain perception at the effector organ with general pain perception. Regarding dental pulp sensibility, dental tests with CO_2_ snow and EPT were applied. The results showed no significant differences between SB and non-SB subjects in terms of pain threshold and duration. For the estimation of the general pain perception, the CPT was applied. Three measures were recorded using this test, namely the general pain threshold, the general pain tolerance, and the general subjective pain intensity. While the general pain threshold and the general subjective pain intensity revealed no differences between both groups, the general pain tolerance was significantly reduced in SB subjects. Thinking about the possible reasons, the latter could be influenced by physiological but also by psychological factors. As the entire study sample consisted of healthy adults between 20 and 50 years of age, physiological-somatic mechanisms could not easily explain the lowered general pain tolerance. One might possibly suspect the amount of subjects with painful TMD in the SB group. However, in comparison with previous studies, reporting a frequency of painful TMD in individuals diagnosed to have SB of 46.4% or more^19,21^, the proportion in the present study appears comparatively low and approximately within the range that was found in the general population^24^. Psychological aspects, such as psychological load, are further possible factors, which may have altered the general pain tolerance. Although the data of the SCL-90-R show no significant difference, an exploratory analysis revealed that SB subjects had significantly higher scores on the somatization subscale than did non-SB subjects. The higher general subjective sensibility of the teeth and lowered general pain tolerance of SB subjects can possibly be understood as an expression of somatization tendencies, a very common phenomenon^35^. Increased and overly focused attention on internal somatic stimuli, especially on the supposedly vulnerable effector organ (the teeth), could cause cognitive misinterpretation. This could result in chronic hyper-vigilant pain perception via the process of somatosensory amplification^36^.

The estimation of the dental pulp sensibility included both subjective rating of the general sensibility of the teeth and the standardized measurements of the dental pulp sensibility. The latter included experimental procedures such as EPT and thermal pulp testing with CO_2_ snow. In the present study, SB subjects rated their general subjective sensibility of the teeth significantly more intensely and tended to perceive their subjective dental pain intensity more pronounced after CO_2_ snow application than non-SB subjects. When interpreting these findings, currently available knowledge on the pulp–dentin complex needs to be taken into account. Classically, secondary dentin is produced throughout the lifespan. Moreover, the pulp–dentin-complex responds to peripheral stimuli with sclerosis of the dentine tubules to reduce their permeability and secretion of tertiary dentine in the direction of the affecting stimulus^29,30^. Both are aimed at protecting the dental pulp from possible noxious stimuli. Consequently, a reduced tooth sensibility would be expected. If these mechanisms will be transferred to a masticatory system with general attrition, it seems likely from a physiological point of view that such subjects would exhibit generally reduced tooth sensibility. However, the results of this pilot study reveal different findings concerning the dental pulp sensibility. From a purely descriptive standpoint, the response of SB subjects was more pronounced than that of non-SB subjects on each of the five tests of tooth pulp sensibility, with one test statistically significant and one trending. Thus, it seems that this discrepancy could not be easily clarified by the physiological reaction of the pulp–dentin-complex. Other possible mechanisms that might influence or modify the dental pulp sensibility, such as psychosomatic factors, appear worth to be reflected.

The subjectively perceived low oral health-related quality of life of SB subjects, assessed by the OHIP-G-14, is comparable to that of patients with complete dentures without treatment needs^37,38^. In the present study, SB subjects showed a significantly higher value in the OHIP-G-14 sum score, which indicates a worse oral health-related quality of life. This is also reflected in the descriptive data of the specific domains, since SB subjects revealed higher values in every domain. This is in line with other studies, which report significantly higher values in the OHIP-14^39,40^. On the other hand, da Silva and colleagues found no association between possible SB and oral health-related quality of life, with the lack of significance possibly due to unequal group distribution (SB, n = 6; non-SB, n = 30)^41^. This comparison highlights a possible negative relation to SB subjects’ own masticatory system. It can be assumed that subjects with SB have some knowledge about SB and its putative negative effects on the effector organ, such as the teeth. Consequently, an increased cognitive engagement on the masticatory system paired with concerns about progression of tooth damage could contribute to an enhanced pain perception. Our findings, an increased general subjective sensibility of the teeth and trend to a stronger subjectively perceived dental pain intensity following to CO_2_ snow testing, support this idea. Poluha and colleagues also reported that catastrophizing pain increases the chance of temporomandibular joint (TMJ) pain in subjects with TMJ clicking^42^. This phenomenon might also be explained by the principle of somatosensory amplification.

A representative sample of 105 healthy subjects and approximately evenly distributed groups was obtained, increasing the generalizability of the results. However, the exclusion of almost 20% of the subjects from the initial sample due to dental criteria must be viewed critically, so that a selection bias cannot be ruled out. Otherwise, this percentage of excluded participants appear little surprising. Many potential participants have been unaware of crowns, root canal filled teeth and other relevant reasons leading to an exclusion from participating in the present investigation. The use of standardized methods for measuring pain allows future replication studies to be conducted.

The present study has some methodological shortcomings that need to be addressed. First, in the present study, the diagnosis of probable SB was based on the criteria presented by the international consensus on the assessment of bruxism^1^. Since probable SB was not determined by instrumental methods, a selection and interpretation bias may be present. The distribution of SB and non-SB might have changed if instrumental methods such as portable EMG devices had been used^43^. However, this diagnostic method for assessing SB was chosen, because it was applicable for the inclusion of a larger sample and according to international consensus reports^1^. It allows the assessment of probable SB. Indeed, the application of the highest standard, the polysomnographic recordings including audio and video, would have permitted the diagnosis of definite SB. However, as discussed in detail elsewhere, the polysomnographic recordings are not suitable for the evaluation of larger sample sizes or studies with sophisticated designs due to its timely, financial, and technical complexity^2,3^. Furthermore, it should be noted that the variance of data on psychological load is limited due to the predefined exclusion criteria. The absence of psychiatric diseases was a prerequisite for participation in the study. Future studies could in turn explicitly examine the association between psychological or psychosomatic illnesses and an altered pain perception in SB subjects. Classification systems such as the International Statistical Classification of Diseases and Related Health Problems in its 10th revision (ICD-10)^44^ or the Diagnostic and Statistical Manual of Mental Disorders in its 5th revision (DSM-5)^45^ should be used. Furthermore, somatization tendencies are worth to study with screenings or interviews. Despite of the limitations, the pilot study comprised a large sample size. So, the results might give a meaningful impression of pain perception and pulp sensibility in SB. The feasibility is largely fulfilled, given the availability of the experimental procedures presented.

## Conclusions

The measurement of general pain perception and dental pulp sensibility with experimentally induced pain is in part a feasible technique to study pain perception in probable SB subjects. Objectively measured pain showed no all-encompassing differences between probable SB and non-SB subjects. Differences were limited to single measures of pain perception such as general pain tolerance. However, the pilot study revealed that probable SB subjects subjectively rated their teeth as being more sensible and tended to perceive dental pain more intense than non-SB subjects. The poorer oral health-related quality of life and higher scores on somatization in probable SB suggest that pain in probable SB is potentially modulated by psychological parameters. A somatosensory amplification could explain the stronger pain perception. It is recommended to focus on somatization tendencies or other psychological vulnerability factors when studying pain in SB subjects.

## Methods

### Study design and blinding

This monocentric pilot study with a parallel group design (SB subjects vs. non-SB subjects) was conducted from March 2013 until June 2016 in the Department of Operative Dentistry, Periodontology, and Endodontology (Heinrich-Heine-University, Düsseldorf, Germany). The experimental procedure involved the performance of various pain-inducing procedures to investigate both general pain perception and dental pulp sensibility. The study conception included two appointments. At the first appointment, inclusion and exclusion criteria have been proven. One trained dentist verified the criteria for assessing probable SB^1^. This allowed the examination of a larger sample^4^. Furthermore, as part of the clinical dental examination, each subject was also scrutinized for signs and symptoms of temporomandibular disorders (TMD) according to the procedures suggested by the Research Diagnostic Criteria for TMD (RDC/TMD) by the same experienced dentist^46,47^. The focus of the present investigation was to examine the differences regarding the general pain perception and the dental pulp sensibility in SB subjects and non-SB subjects. The presence or absence of a TMD diagnosis according to the RDC/TMD was recorded as a co-variable, not being an exclusion criterion. In doing so, authors aimed for collecting a more naturalistic sample. As known from the literature^19,21^, SB samples included a mean percentage of approximately 50% with a TMD diagnosis. On the second appointment, another trained dentist, blinded to the SB diagnosis, performed the pain-inducing procedures to rule out investigator bias. The present report is in line with the Strengthening in the Reporting of Observational Studies in Epidemiology (STROBE) statement^48^.

### Study sample

Participants were recruited from patients seeking treatment at the University Hospital of the Heinrich-Heine-University of Düsseldorf. They were also able to respond to study information on the department's website or posters on campus. Participants were all German native speakers. All subjects gave informed consent to the procedures. Based on recommendations^49^, a size of at least 30 subjects per group were chosen. Considering a dropout rate of 20%, the desired minimum size of the total sample was set to 72.

Healthy adults between the 20 and 50 years of age were included in the study. General exclusion criteria, which would exclude a healthy general condition, were: severe mental disorder, abusive use of or a dependence on drugs or medications, central nervous system and/or peripheral nervous system disorders, as well as other severe physical or systemic diseases, such as cardiovascular disease, autoimmune disease, respiratory insufficiency, or an active inflammation or malignant disease, which were assessed by anamnestic questionnaire. Pregnant and breastfeeding women were also excluded from participation in the study. Further dental exclusion criteria were: more than two missing molars (excluding third molars), the presence of prosthesis or extensive prosthetic restorations, fixed orthodontic treatment, current application of dentine desensitizing agents onto teeth, and the presence of gross malocclusion (i.e. anterior open bite). For the tooth, which was subjected to the electrical and thermal pulp testing, further dental exclusion criteria were: negative tooth sensibility, carious lesions, cervical restorations, crowns, and the presence of a pulpitis on the test tooth and/or the adjacent teeth, and insufficient and/or extensive occlusal restorations.

Based on the diagnostic criteria of the international consensus report^1^, the inclusion criteria for the probable SB group were anamnestic information (e.g., report of the bed partner hearing the patient grind their teeth at night) as well as clinical features (e.g., masticatory muscle hypertrophy, attrition)^4,50,51^. Participants from whom SB could be excluded represented the non-SB control group.

### Measures

#### General pain perception

**Figure 2:**
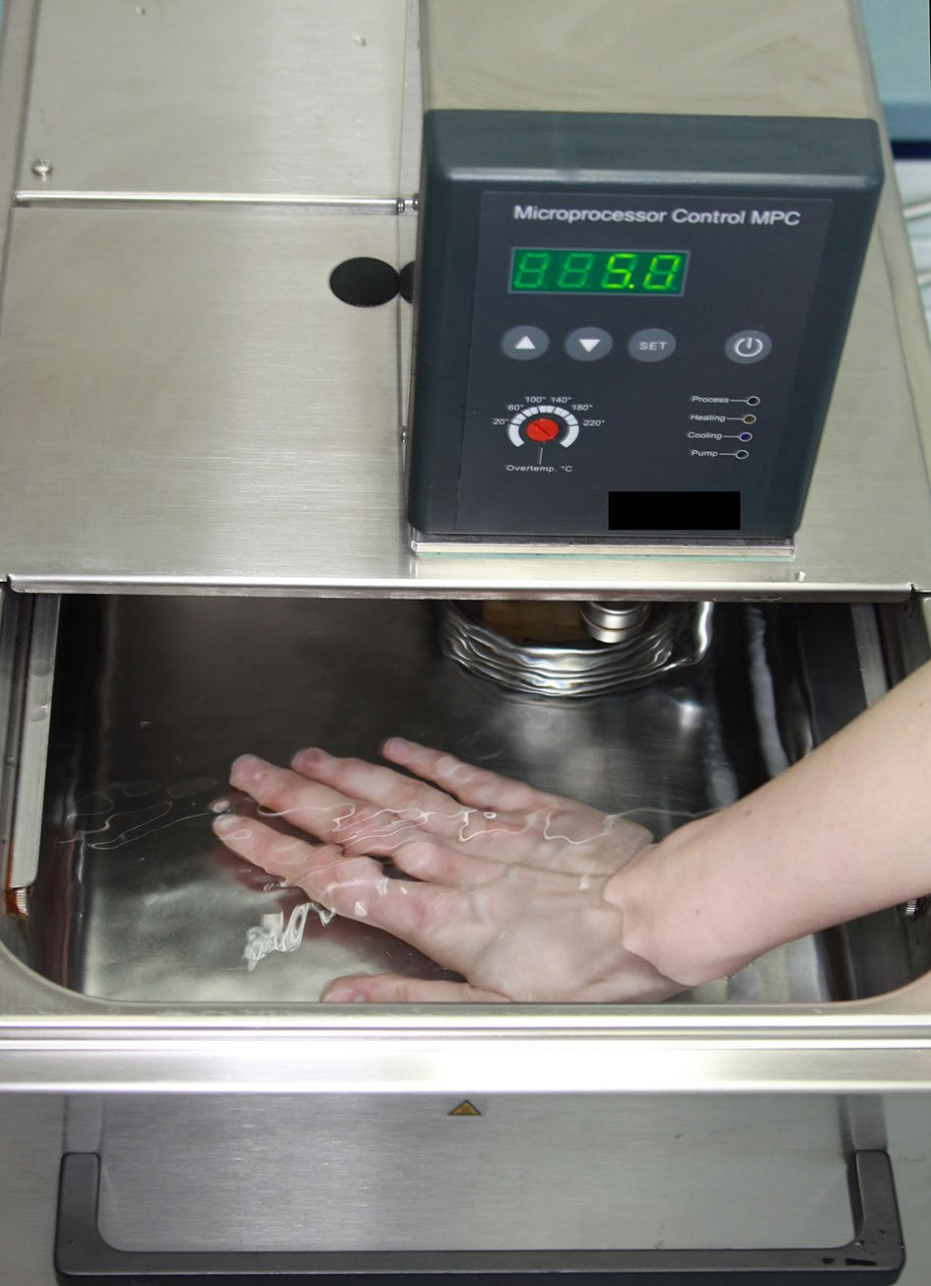
Circulating water bath system for the cold pressor test with immersed right hand. The water temperature was a constant 5 °C throughout the basin.

The cold pressor test (CPT) was used to assess general individual pain perception (Fig. 2). In this standardized setting, the CPT was performed with a circulating water bath system (MPC-208B; Huber Kältemaschinenbau, Offenburg, Germany). It includes a specific cooling mechanism and water circulation, so that the water temperature in the entire basin was 5 °C^52–54^. As described in previous studies^55,56^, participants were instructed to place their hand in the cold water up to the wrist. Simultaneously, the examiner started an electronic time clock (JS-9004, Conrad Electronic, Hirschau, Germany). When participants first felt a pain sensation, they signaled the examiner to stop the timer by raising the other hand. Participants were asked to hold their hand underwater until they could no longer tolerate the pain. Immediately after removing the hand from the basin, participants were asked to rate their pain intensity. It was indicated on a visual analogue scale (VAS) ranging from zero (no pain) to 100 mm (worst pain imaginable). During the CPT, the following three parameters were measured: (1) *General pain threshold* was assigned as the amount of time until the participant reported the first pain sensation; (2) *General pain tolerance* was operationalized as the amount of time between the beginning of the test and the removal of the hand from the cold water; (3) *General pain intensity* was defined as the subjectively perceived degree of pain intensity assessed using the VAS as described above. For safety reasons, each participant was informed that the CPT would be terminated after five minutes, even if they had not reached yet their maximum pain tolerance^47^.

#### General subjective sensibility of the teeth

During the first appointment, the degree of the general subjective sensibility of the teeth was estimated. Participants were asked to answer the question “In general, are your teeth particularly sensitive?” using a numeric rating scale (NRS) ranging from zero (not at all) to ten (very much).

#### Dental pulp sensibility

**Figure 3:**
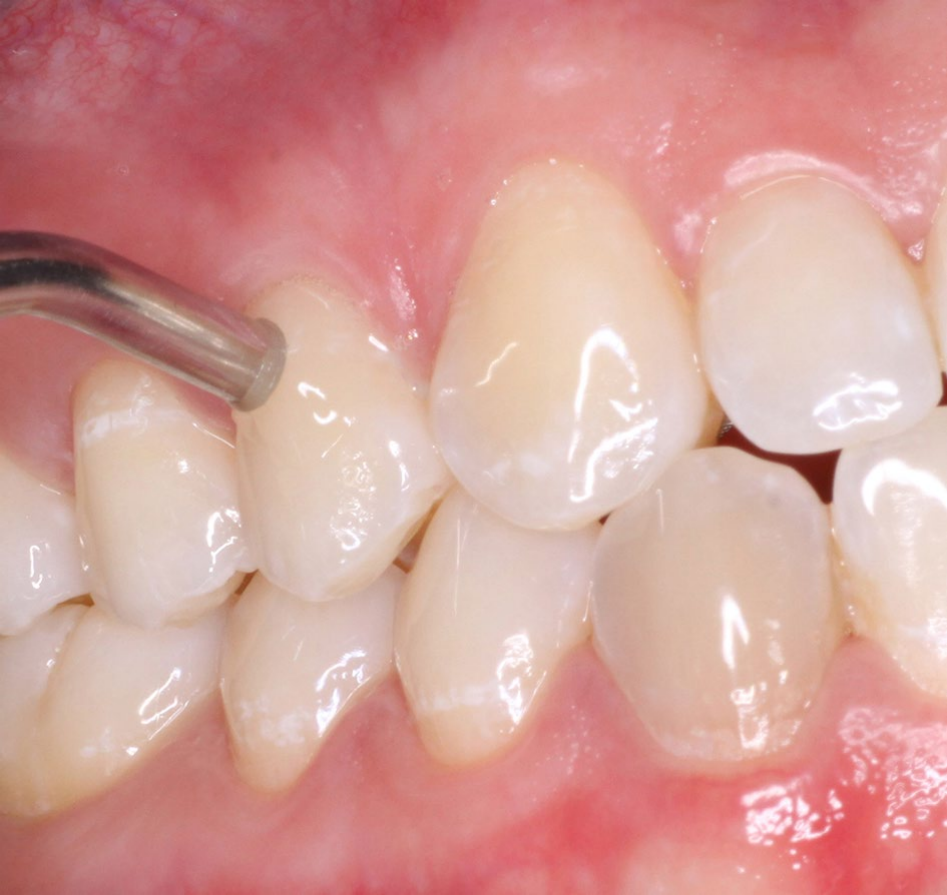
Application of the electrode tip onto the buccal test tooth surface using fluoride gel as conduction medium.

The estimation of the dental pulp sensibility included electrical as well as thermal pulp sensibility tests. The selection of the test tooth was based on the following order: first premolars, second premolars, first molars, preferably in the maxilla in each case. The former test was performed by means of the electric pulp test (Vitality Scanner 2006; Kerr, Brea, CA, USA) according to the manufacturer’s recommendations. The test tooth was isolated with cotton rolls and dried. After that, the electrode tip was positioned in the middle third of the buccal tooth surface using fluoride gel as conductive medium (Fig. 3)^57–59^. When the probe contacted a tooth, the electric pulp test (EPT) automatically turns on. After the unit turned on, the intensity of the electrical stimulus increased automatically. The rate of the stimulus rise could be selected between slow and fast. The voltage was set to medium level increase throughout the study as reported by other investigators^59^. Participants were asked to immediately indicate whether they felt a tingling sensation in the test tooth by raising a hand. At the same moment, the probe was lifted from the tooth and the stimulus level was recorded on the display, viz. the *EPT-unit*. A negative response rate was documented when a value of 80 EPT-units was reached and the participant realized no pulpal sensibility. As derived from the manufacturer’s recommendations, the normal response ranges for the EPT-units of premolars were between 20 and 50.

**Figure 4:**
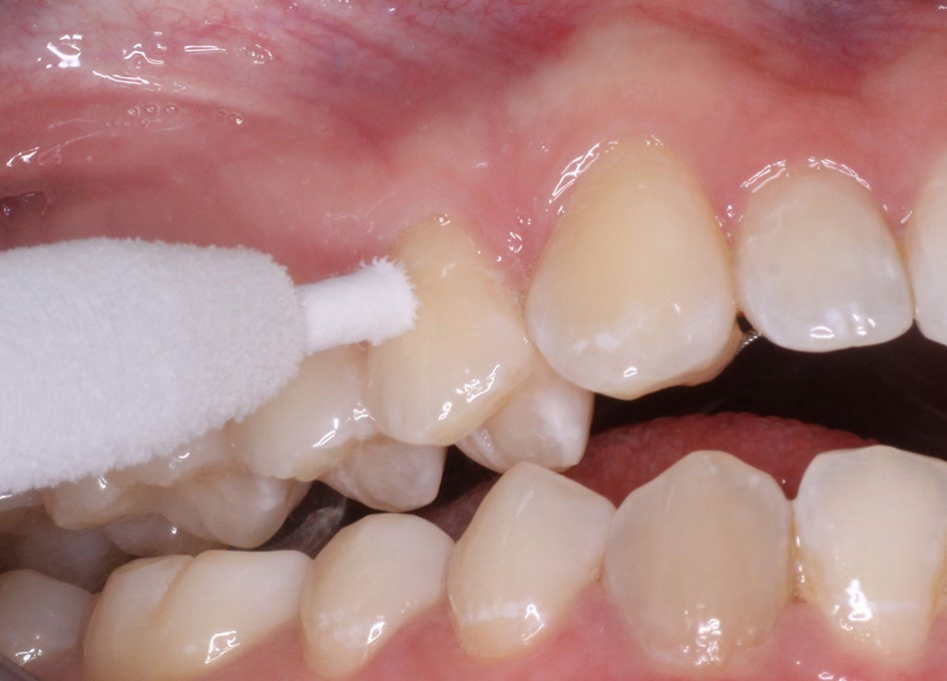
Application of a CO_2_ snow stick onto the buccal surface of the test tooth.

The thermal pulp sensibility test was conducted with CO_2_ snow formed into a CO_2_ stick by using a specific adapter (Fig. 4). This allowed the CO_2_ crystals to be compressed into a cylinder of approximately 3.5 mm diameter (Odontotest; Contrag, Zürich, Switzerland). The test tooth was again isolated with cotton rolls and dried. After that the CO_2_ stick was applied in the middle third of the buccal tooth surface^57–59^. At this moment, an electronic time clock button was pressed. As mentioned earlier^59,60^, participants were instructed to raise a hand when they first felt a moderate pain. The CO_2_ stick was then immediately removed from the tooth surface. The application time thus determined represented the *dental pain threshold*. They were further advised to give a hand signal when the pain has been completely subsided (*dental pain duration*). The electronic time clock button was then pressed again. Immediately after the CO_2_ test, subjects quantified their *subjective dental pain intensity* by using a VAS. Similar to previous studies, the duration of the CO_2_ application was limited to 15 s^59^. If no response was observed during that period, a negative response was recorded and the subject had to be excluded. After the application of EPT or CO_2_ snow on the tooth surface, a rest period of two minutes was considered to allow the pulpal border of the dentin to return to normal temperature^59^.

In order to omit a possible bias resulting from the order of test application (CPT and dental pulp testing), the order was permuted. For this, after each patient the sequence of the applied tests has been changed (A: first CPT and second dental pulp testing or B: first dental pulp testing and second CPT).

### Psychometric instruments

The severity of psychological load was measured by two self-evaluation questionnaires. The German version of the Symptom-Checklist-90-Standard (SCL-90-R) was applied, to assess impairments caused by physical and psychological symptoms^34^. This self-report instrument consists of nine scales: somatization, obsessive compulsive, interpersonal sensitivity, depression, anxiety, hostility phobic anxiety, paranoid ideation and psychoticism. Subjects answered 90 items on a five-point Likert scale, ranging from zero (not at all) to four (extremely). The indication refers to symptoms they suffered from during the last 7 days. To assess the level of psychological load, data were summarized into the Global Severity Index (GSI; interval-scaled value from zero to four). High scores represent higher psychological load. Regarding the nine scales of the SCL-90-R, raw data were summed for each scale and divided by the number of items per scale to a mean value (in each case an interval-scaled value from zero to four).

The oral health-related quality of life was measured with the German version of the Oral Health Impact Profile (OHIP-G-14), which consists of 14 items^37^. The items refer to the period of the last month and focus on difficulties and problems with, for example, speech production and food intake. Information about the frequency of their occurrence is given on a five-point Likert scale from zero (never) to four (very often). Data were summarized to a total sum, ranging from 0 to 56. High scores indicate poor oral health-related quality of life. For the interpretation of the data, reference values of different patient groups without treatment need are available: Data from 50% of the participants with natural teeth without removable partial prostheses reported a sum score of 0 (95% CI 0–0), 50% of the participants with removable partial prostheses reported a sum score of ≤ 4 (95% CI 2–5), and 50% of the participants with complete dentures reported a sum score of ≤ 6 (95% CI 4–11)^38^. Furthermore, the four dimensions of the OHIP-G-14 were evaluated, which capture oral function, orofacial pain, orofacial appearance, and psychosocial impact. The version with four dimensions was selected, as it fits the data better instead of the known version with seven dimensions (functional limitations, physical pain, psychological discomfort, physical disability, psychological disability, social disability, and handicap)^61^.

### Outcome

The feasibility of the study was evaluated based on the following criteria: Recruitment, retention and refusal rates and determining capacities like process time and challenges with experimental procedures. The primary outcome were pain-related and the secondary outcome psychometric variables. The first are measured either standardized via CPT, EPT or thermal pulp test or subjectively via self-assessment scales. Standardized pain-related outcome variables were the general pain threshold (s), the general pain tolerance (s), the number of EPT units, the dental pain threshold (s), and the dental pain duration (s). Subjective pain-related outcome variables were the general subjective pain intensity (VAS; mm), the general subjective sensibility of the teeth (NRS; interval-scaled value from zero to ten), and the dental subjective pain intensity (VAS; mm). Regarding the psychometric properties, the GSI (interval-scaled value from zero to four) and the mean values of the nine subscales (in each case interval-scaled value from zero to four) of the SCL-90-R were considered. Furthermore, the individual sum of items of the OHIP-G-14 (interval-scaled value from 0 to 56) and the individual interval-scaled dimensions, oral function (score from 0 to 20), orofacial pain (score from 0 to 4), orofacial appearance (score from 0 to 4), and psychosocial impact (score from 0 to 28), was included in the statistical evaluation.

### Statistical analysis

First, general sample characteristics were examined by descriptive methods. For location parameters, the mean and standard deviation were chosen for interval-scaled and normally distributed data. For non-normally distributed data, the median is depicted. Distribution of nominal-scaled data are presented as absolute and relative frequency. Data were checked visually via QQ-plots and Shapiro–Wilk test for normality. In order to rule out systematic group differences concerning confounding variables, sociodemographic and descriptive data were examined prior with *t*-tests (or non-parametric alternatives like Mann–Whitney *U* test) or Chi-square test for nominal data. A one-factorial multivariate analysis of variance (MANOVA) was computed. Assumptions for performing a MANOVA, such as the presence of homoscedasticity, were checked with appropriate tests (e.g., Levene’s test). The diagnosis of SB (SB vs. non-SB) served as the independent group variable and eight pain-related measures as outcome variables. As the omnibus test revealed a significant result, post-hoc univariate analyses of variance (ANOVAs) were conducted to determine significant differences for each dependent variable. For the psychometric data, parametric or non-parametric tests for comparing means between SB and non-SB subjects were performed. The alpha-error rate was set to *p* = 0.05 for every test. Corrections for multiple comparisons beside the main statistical analysis were made by controlling the false discovery rate^62^. All statistical analyses were performed with the data analyzing language R and its appropriate software (RStudio Desktop; RStudio, Boston, MA, USA).

### Ethics declarations

All procedures performed in studies involving human participants were in accordance with the ethical standards of the institutional and/or national research committee and with the 1964 Helsinki Declaration and its later amendments or comparable ethical standards. The study was approved by the Institutional Human Subjects Ethics Committee of the Medical Faculty of the Heinrich-Heine-University of Düsseldorf (No. 3832).

### Informed consent

Written informed consent was obtained from all participants included in this study. No identifying information is included in this article.

## Data Availability

The datasets generated during and/or analyzed during the current study are available from the corresponding author on reasonable request.
